# Low-Cost and Highly Sensitive Pressure Sensor with Mold-Printed Multi-Walled Carbon Nanotubes Dispersed in Polydimethylsiloxane

**DOI:** 10.3390/s21155069

**Published:** 2021-07-27

**Authors:** Tim Mike de Rijk, Walter Lang

**Affiliations:** Institute for Microsensors, Microactuators and Microsystems (IMSAS), University Bremen, 28359 Bremen, Germany; WLang@imsas.uni-bremen.de

**Keywords:** carbon nanotube, percolation threshold, screen-printing, mold-printing, pressure sensor, conductive polymer, numerical models

## Abstract

Flexible pressure sensors with piezoresistive polymer composites can be integrated into elastomers to measure pressure changes in sealings, preemptively indicating a replacement is needed before any damage or leakage occurs. Integrating small percentages of high aspect ratio multi-walled carbon nanotubes (MWCNTs) into polymers does not significantly change its mechanical properties but highly affects its electrical properties. This research shows a pressure sensor based on homogeneous dispersed MWCNTs in polydimethylsiloxane with a high sensitivity region (0.13% kPa${}^{-1}$, 0–200 kPa) and sensitive up to 500 kPa. A new 3D-printed mold is developed to directly deposit the conductive polymer on the electrode structures, enabling sensor thicknesses as small as 100 μm.

## 1. Introduction

**Figure 1:**
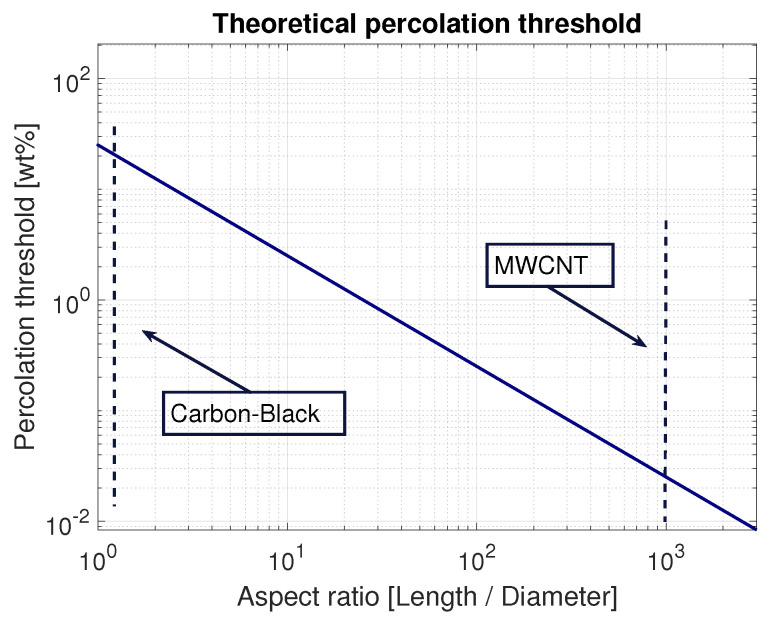
Theoretical percolation threshold according to the equation from Chen et al. [17]. Carbon-Black particles have an aspect ratio of 1, and carbon nanotubes are several orders higher.

Interest in flexible electronics keeps increasing. Standard silicon-based sensors become interchanged with flexible substrates and conductive polymers. Fillers are added to enhance mechanical, electrical or thermal properties of the polymer. Adding specific amounts of metallic particles can alter the polymer to become conductive. Achieving percolation depends highly on the fillers aspect ratio (AR) and specific surface area. Metallic powders often need a concentration of up to 60% to achieve percolation [1]. Carbon-black nanoparticles decrease this threshold to a reported range of 3 to 15 wt% [1,2], which was demonstrated in our previous study, where a screen-printed resistive pressure sensor based on carbon-black particles was manufactured [3]. To decrease the filler content and minimise the change in mechanical properties of the polymer, carbon nanotubes (CNTs) can be integrated, resulting in a percolation threshold of at least one order of magnitude lower than its carbon-black counterpart [1,4] (Figure 1). An extensive review from Wolfgang Bauhofer et al., 2009 [5] showed achieved percolation thresholds as low as 0.035 wt% and up to 2.4 wt%. The reported widespread can be explained by the various processing techniques implemented, and chosen carbon nanotube (CNT) types. The CNTs disperse differently in different (organic) solvents. Several solvents often used to facilitate the dispersion of CNTs in polymers are: Isopropanol [4,6,7], ethanol [7], acetone [8], chloroform [9,10,11,12], Tetrahydrofuran (THF) [13], Toluene [9,14,15] and Dimethylformamid (DMF) [16]. However, if a good CNT dispersibility in a solvent is possible, it is crucial that the solvent dissolves the polymer as well. Solvent examples for this are DMF and Toluene. DMF has a good dispersibility for CNTs; however, it reacts with the PDMS base rasin [9]. Toluene was found to have a great solubility in PDMS but a poor CNT dispersion [9].

Statistically, the percolation threshold should decrease with an increasing aspect ratio. However, the key is to have the CNTs as homogeneous as possible in the layer. Highly physically entangled CNTs cause a large increase in the percolation threshold [1]. Theoretical percolation thresholds can be modelled by looking at the filler interparticle distance in a volume, which implores the homogeneous distribution of filler materials. Due to the lack of entangled clusters of CNTs, they achieve very low theoretical thresholds.

A large variety of techniques are proposed throughout the literature for lowering the percolation thresholds by overcoming the physical entanglement and large van der Waals forces between the Chemical Vapor Deposition (CVD) grown MWCNTs [5,6,14,18]. The most commonly employed methods are shear mixing and ultrasonic treatment, which are both able to break up the agglomerated CNTs [1,9,10,12,16].

However, little research has been conducted to show the theoretical behaviour of pressure sensors with high aspect ratio fillers embedded in a polymer. This study thoroughly investigates the working principle of a pressure sensor with dispersed CNTs and applies this to the manufactured sensors, as well as providing the necessary data to increase sensitivity and pressure ranges.

The aim of this research is to manufacture a low-cost flexible pressure sensor that can be integrated in various elastomers where rigid silicon-based sensors would not be feasible. A new manufacturing technique is demonstrated, facilitating fast and low-cost fabrication of pressure sensitive conductive layers. This method does not rely on a cleanroom with its corresponding expensive equipment and fabrication steps such as lithography, etching and spin-coating. Therefore, the sensors can be manufactured more quickly (no lithography masks needed) and with less cost than their corresponding cleanroom-manufactured counterparts. This type of sensor could be integrated into O-ring sealings, monitoring shifts in pressure and indicating elastomer failure [19].

## 2. Materials and Methods

### 2.1. Polymer Composite

**Figure 2:**
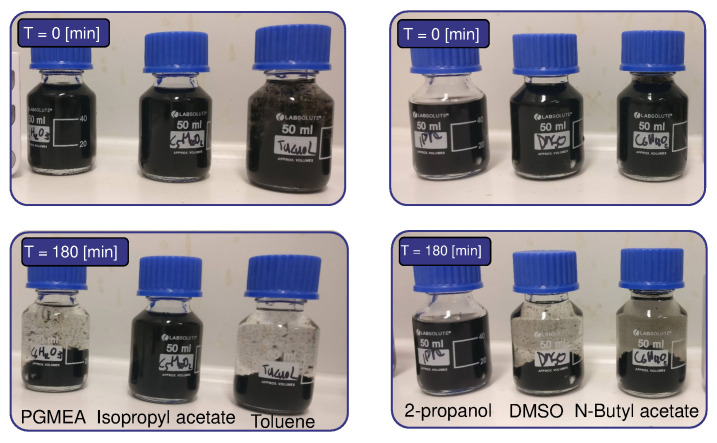
Solvents mixed with multi-walled carbon nanotubes. From left to right: PGMEA, Isopropyl acetate, Toluene, 2-Propanol, DMSO and N-Butyl acetate.

Polydimethylsiloxane (PDMS, Slygard 184, DOW, USA) is chosen as a flexible polymer because of its well-known properties and widespread adaptation in flexible sensor manufacturing [4,20]. Multi-walled Carbon Nanotubes (MWCNTs) were bought from Ioletic Germany with OD <10 nm, L = 5–15 μm. To improve dispersion of the highly agglomerated MWCNTs, different solvents were tested (Sigma-Aldrich, Taufkirchen, Germany): 2-Propanol (IPA), Isopropyl acetate, Propylene glycol methyl ether acetate (PGMEA), Toluene, Dimethyl sulfoxide (DMSO), Acetone, Ethanol and N-Butyl acetate. After 30 min of mechanically stirring the solutions (700 min${}^{-1}$) and 30 min in an ultrasonic bath, the solutions were placed on a table and monitored over a period of 180 min (see Figure 2). After this period of time, IPA and isopropyl acetate did not visibly change. The solution remained non-transparent, and no settling at the bottom of the vials was detected. All other solutions settled on the bottom of the vials after several minutes. Due to the wide availability of IPA and the good solvability in PDMS, IPA was chosen to be a suitable solvent for dispersing the MWCNTs in PDMS.

The MWCNTs were mixed (700 min${}^{-1}$) for 30 min in IPA (1:100 ratio), after which they were placed in an ultrasonic bath for 30 min to promote de-agglomeration. The PDMS viscosity was decreased by adding IPA (1:6 ratio) and mixing it for 10 min, after which the diluted PDMS was merged together with the CNT solution. The IPA solvent was slowly removed by placing the solution on a hotplate set to 100 °C for 3.5 h (at 250 min${}^{-1}$), after which the solution was cooled by placing it in a cold-water bath before adding the curing agent (1:10). After brief manual mixing, the solution was placed in a vacuum chamber for 30 min to extract trapped air bubbles and ensure removal of all IPA solvent. Immediately after, the MWCNT/PDMS mixture was deposited on the interdigital electrodes (IDEs).

### 2.2. Sensor Principle

The resistive pressure sensor is equipped with interdigital electrodes, and a pressure-sensitive layer deposited on top. External pressures cause the layer to compress, reducing the filler–filler distances of the conductive particles in the polymer and increasing the layer conductivity. This behaviour can be analytically presented in Equation (1), which relates the applied stress σ to the Compressive Modulus *E* [21].

(1) $$s=s_{0}\left(1-\varepsilon \right)=s_{0}\left(1-\frac{\sigma }{E}\right)$$

This relation illustrates that a certain applied stress σ causes the interparticle distance *s* to decrease. The percolation threshold of a material is defined where the resistance suddenly decreases, changing the material from an insulator to a conductor. By mixing high-aspect-ratio metallic particles, such as CNTs, it is possible to greatly decrease this threshold. Although studies show a large range of achieved percolation thresholds, general estimations can be made to approximate filler concentrations needed for achieving conductive paths between electrodes.

**Figure 3:**
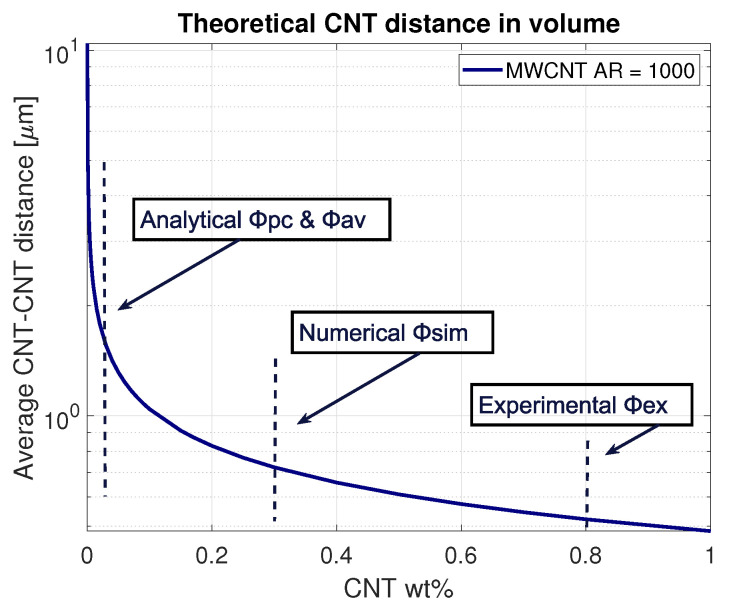
The theoretical average CNT interparticle distance for MWCNTs with AR = 1000. Analytical results ($\Phi_{pc}$ and $\Phi_{av}$) show a percolation threshold of 0.025 wt%. Numeral simulations give $\Phi_{sim}$ = 0.15 wt%. Due to the agglomeration effects, experimental results give $\Phi_{pc}$ = 0.8 wt%, corresponding to the results from Li et al. [1] with 10% agglomeration volume.

The average CNT–CNT distance in a volume for the MWCNT applied for this research was determined by determining the occupied volume of the filler material within the total volume and determining the average distance between them. The result is shown in Figure 3. It can be noticed that the ideally dispersed CNT–CNT distance drops already at very low filler content ($\Phi_{av}=0.02$ wt%).

Chen et al. [17] estimated the percolation threshold to be

(2) $$\Phi_{pc}=\frac{\left(3+\delta_{d}^{2}\right)}{6AR}$$

Here, $\delta_{d}$ is the dimensionless range of diameter: $\delta_{d}=\left(d_{2}-d_{1}\right)/\left(d_{2}+d_{1}\right)$, with the assumption of normalised CNT diameter distribution. Assuming the relation of high-aspect-ratio MWCNT vol% = 2 wt% [1], this relation gives a percolation threshold of $\Phi_{pc}$ = 0.025 wt%.

**Figure 4:**
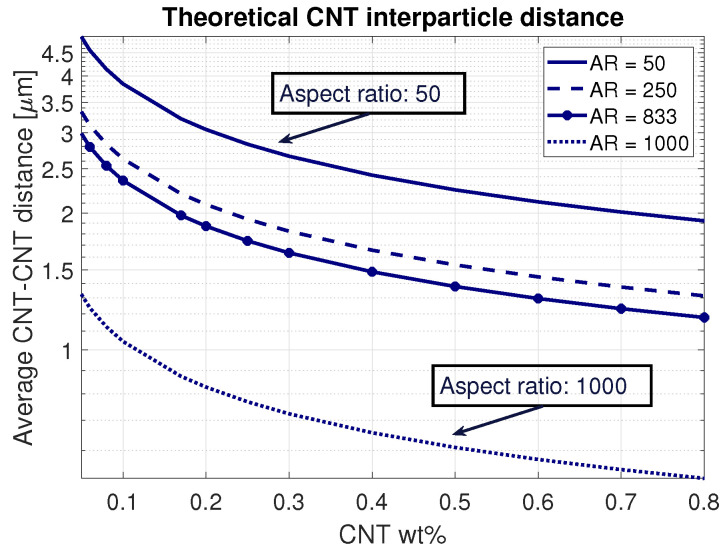
The theoretical CNT interparticle distance for different multi-walled carbon nanotubes in relation with the weight percentage in the polymer. The aspect ratio (AR) influences the percolation threshold due to the large change in CNT interparticle distances.

Both methods do not account for the agglomeration of the carbon nanotubes. Li et al. [1] evidenced that even a CNT agglomeration volume of 5% could already increase the percolation threshold by one order of magnitude. Increasing the volume fraction of agglomerated CNTs to 10% gives a theoretical percolation threshold of 0.8 wt% [1], which is similar to the percolation threshold found in this research. The analytical $\Phi_{av}$ and $\Phi_{pc}$, numerical $\Phi_{sim}$ and experimental thresholds $\Phi_{ex}$ are included in Figure 3 to illustrate the differences between the ideal percolation thresholds and the experimental results. Key parameters influencing these differences are the agglomeration percentage and CNT dimensions. The impact on the latter is exhibited in Figure 4, illustrating the CNT interparticle distances for four different multi-walled carbon nanotubes, depending on the weight concentration. With an increasing aspect ratio, the CNT interparticle distance decreases, which, in turn, increases the chance of creating a conductive polymer. A known difficulty with CNTs is that the chance of entanglement increases with the increased aspect ratio, leading to higher dispersion difficulties. Following these results, it was chosen to obtain CNTs in the range of AR = 700–1000.

### 2.3. Sensor Dimensions

The IDE structures were directly screen-printed (Pröll services GmbH, type 54–64 μm) on 3 mm thick Poly(methyl methacrylate) (PMMA) sheets with a silver paste (Sun Chemical CHSN8013). Dry-layer thicknesses were measured to be 5 μm. The IDEs had a linewidth of 300 μm, gap size of 600 μm and a total area of 11 × 7 mm${}^{2}$, and the pressure-sensitive layer was directly printed on top of it. It is not expected that the sensor behaviour would vary when the sensor is constructed on a flexible or rigid substrate.

## 3. Numerical Results

**Figure 5:**
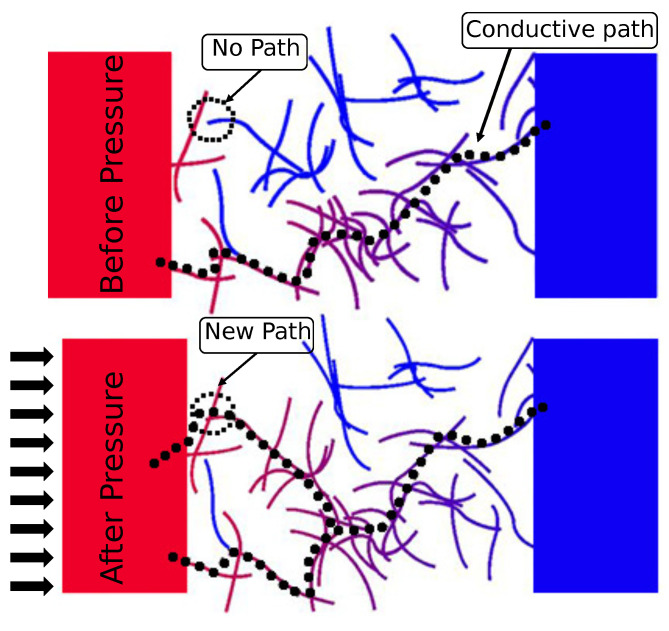
2D Model of randomly dispersed CNTs between two electrodes. For clarification purposes, the CNTs are not drawn to scale in this figure.

A 2D model was created featuring two electrodes with random dispersed MWCNTs in between, as shown in Figure 5. Because the MWCNTs can curve and bend due to its high aspect ratio, the nanotubes cannot simply be modelled as straight long cylinders. Therefore, a Java plugin in COMSOL Multiphysics was implemented to create curved cylinders with an average length of 10 ± 5 μm, identical to the MWCNTs from this study. Random placement and orientation were achieved by rotating the nanotubes with a randomly determined angle between 0 and 360° and placing the tubes randomly in a predetermined grid. The MWCNTS were given a fixed thickness of 10 nm.

In order to give the modelled carbon nanotubes this thickness, the efficient approximate Bézier curve offsetting algorithm by Brouwer and Wilco [22] was integrated. Bézier curve offsetting goes beyond copying the curve and translating its control points linearly since Bézier curves are parameterized.

To quickly calculate the coordinates needed to obtain an approximation of an offset curve, the aforementioned curve offsetting algorithm mimics the approach by Tiller and Hanson [23] whilst avoiding the expensive line–line intersection calculations. This is made possible by the exploitation of several geometric properties of the problem. The distance to the parallel curve and, thus, the thickness of the modelled carbon nanotubes are easily controlled by a variable in the algorithm.

**Figure 6:**
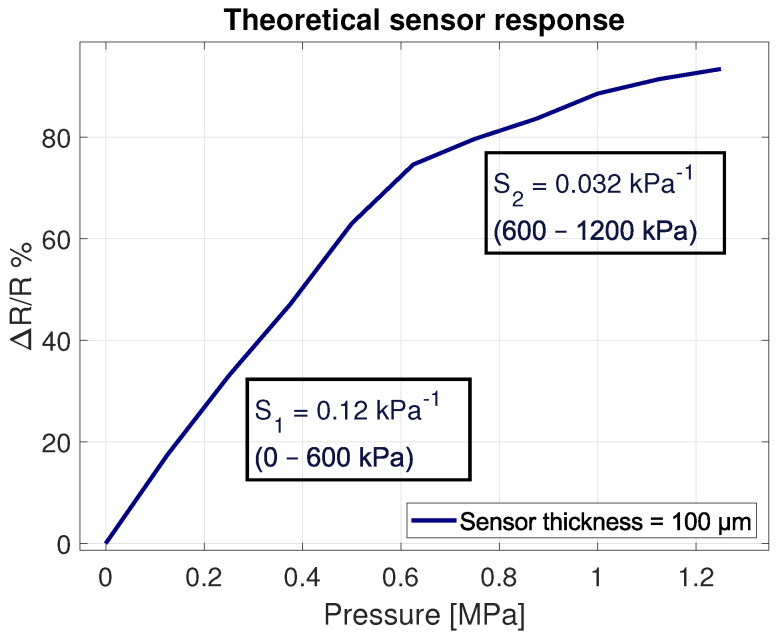
The theoretical sensor response from 2D sensor model.

The chance of conduction was determined and, depending on layer thicknesses, found to be $\Phi_{sim}$=0.30 wt% (measured at 50% conduction possibility). Differences between the theoretical thresholds from Equation (2) and the simulation could be explained by the fact that only a small part of the IDE was simulated (a length of 100 μm). It is expected that increasing the length results in higher conductivities at lower concentrations, closing the gap between the analytical approach and the numerical simulation. However, increasing the simulation area exponentially increases the computation time due to the added number of carbon nanotubes in the volume. An increasing force is applied to one side of the sensor. The response is presented in Figure 6. Two clear regions can be identified: The first region (0–600 kPa) reveals a highly sensitive region ($S_{1}$ = 0.12% kPa${}^{-1}$), whereas the second region (600–1200 kPa) shows a slight decrease in sensitivity ($S_{2}$ = 0.032% kPa${}^{-1}$).

**Figure 7:**
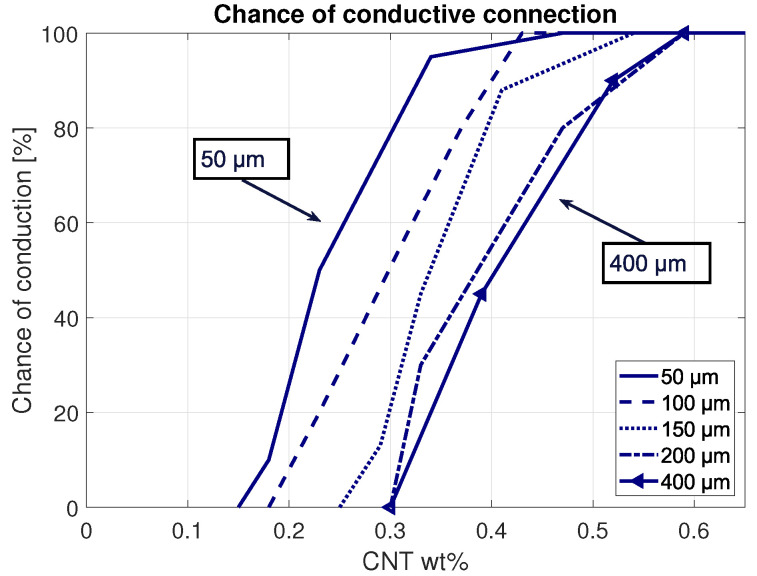
Statistical chance of formed conductive networks between electrodes with different sensor thicknesses.

**Figure 8:**
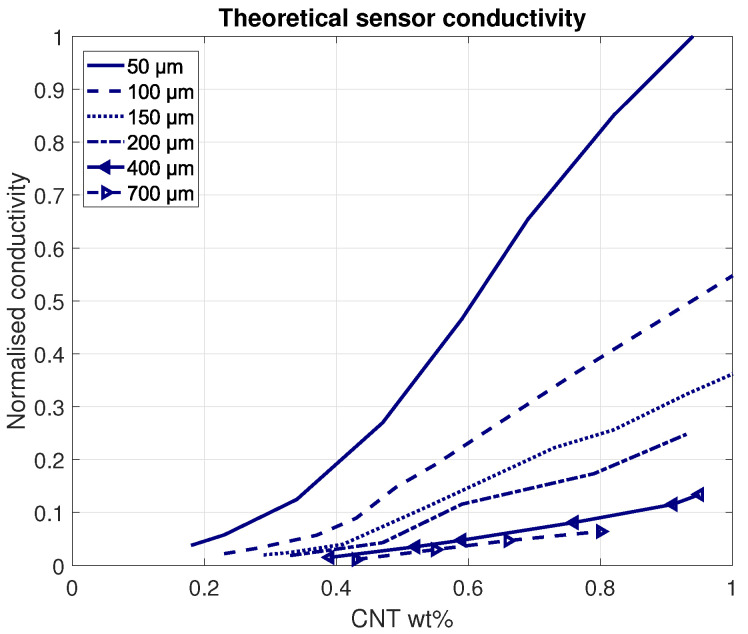
The theoretical conductivity for different sensors thicknesses. The thicker the layer becomes, the less sensitive the sensor.

Different layer thicknesses (50–700 μm) were simulated to find the effect on the sensor response. Conductivity in the thin sensing layers are expected to change rapidly, as the layer is in the same order of magnitude as the lengths of the CNTs. With increasing thickness, it takes relatively more nanoparticles to overcome the longer paths between the electrodes. For example, a sensor with 50 μm thickness and 10 μm CNTs needs at least five consecutive CNTs to form a conductive path between both electrodes. This number increases with the increasing sensor thicknesses. In turn, the chance of such long connective paths decreases with the increasing sensor thickness (as presented in Figure 7). Applying a directional force to one side of the sensor with a fixed weight percentage of carbon nanotubes effectively increases the nanoparticle weight percentage due to the compressing polymer. The results confirming these expectations are displayed in Figure 8, where it can be clearly seen that thinner layers exhibit larger conductivity changes with respect to CNT weight percentages. Hence, it can be argued that thinner layers experience higher sensitivities.

## 4. Experimental Results

**Figure 9:**
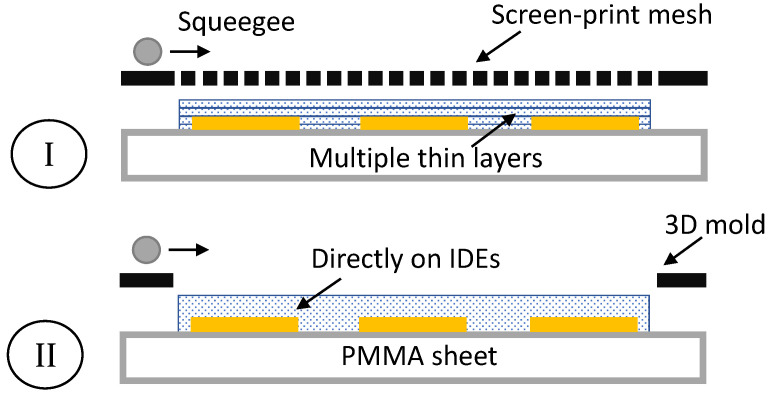
Schematic sensor overview.

Two different manufacturing techniques were exploited for fabrication of the pressure sensitive layer: a screen- and a 3D-mold-printing method, as schematically illustrated in Figure 9.

**Figure 10:**
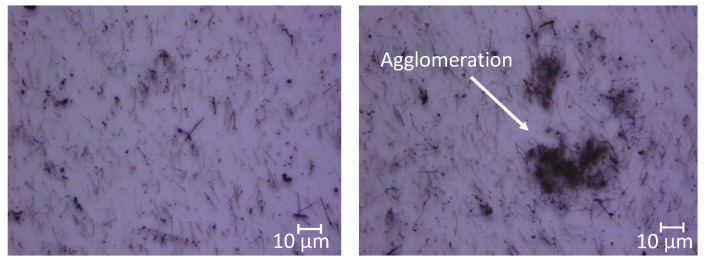
Optical transmission microscope images of 0.5 wt% MWCNT at two different magnifications, showing individual (**left**) and agglomerated (**right**) CNTs.

It was found that compositions with more than 0.8 wt% MWCNT were conductive (measured resistance 2–4 MΩ, thickness 300 μm). Increasing the weight percentages to more than 7% caused the layers to become very brittle. Flexing the material resulted in cracks throughout the surface. Hence, it was chosen to keep the carbon nanotube weight percentages below this threshold. The quality of the nanotube dispersion in the polymer was assessed by looking at optical microscopy images of thin 30 μm layers. This provided an approximate 2D layer of the MWCNT–PDMS composite, where it was possible to determine interparticle distances and volume agglomeration percentages (Figure 10). The results evidenced an average interparticle distance of 6 μm. According to the results shown in Figure 4, ideally dispersed MWCNTs should have an average distance of 0.7 μm. This large difference can be accounted for due to agglomerated CNTs, which occupied an average volume percentage of 10.7%. Considering the CNT aspect ratio (1000) and estimated agglomeration volume, the percolation threshold should lie at 0.8 wt%, according to Li et al. [1], which agreed with the results found in this study.

The relation between AR and CNT dispersibility was investigated by dispersing two identical CNTs with different ARs (CNT${}_{1200}$ and CNT${}_{300}$). The average agglomeration sizes were found to be CNT${}_{300}$ = 18μm and CNT${}_{1200}$ = 38μm (measured with the KEYENCE VK-9700 microscope, KEYENCE Deutschland GmbH). The overall area of large agglomerations (total area/agglomeration areas) did not significantly change with different ARs, showing that higher AR gave fewer but larger agglomerations. Therefore, there could be a trade-off between AR and dispersion quality. The higher the AR, the lower the theoretical percolation threshold can be. However, this increases the difficulty for homogeneous dispersion, effectively increasing the percolation threshold again.

**Figure 11:**
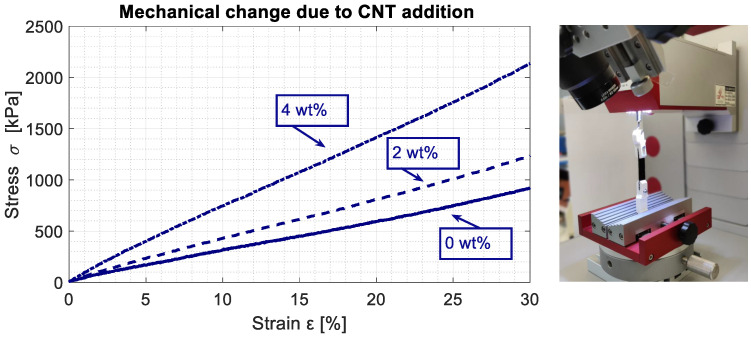
Stress–strain relations for PDMS/CNT samples with different concentrations.

Mechanical properties of the composites were determined by placing samples (0, 2 and 4 wt%) in a bond tester (Condor 100 XYZTEC, XYZTEC Germany). The 130μm thick samples were subjected to a maximum strain of 30%, with a speed of 200 μm. The elastic modulus is determined by the relation E=σ/ϵ, and the results were averaged over four samples. The elastic modulus of pure PDMS was found to be 3 MPa, corresponding with literature values [24]. Increasing the CNT content of the polymer increased the stiffness of the material (E${}_{2wt\%}$ = 4.1 MPa, E${}_{3wt\%}$ = 7.1 MPa). The results and experimental setup of the stress–strain tests are shown in Figure 11.

### 4.1. Screen-Printed Sensitive Layer

A 4 wt% CNT pressure-sensitive layer was deposited via manual screen-printing directly on the IDEs, having a single dry-layer thickness of roughly 30 μm. Several layers were printed on top of each-other. After each consecutive layer, the PMMA sheet was placed in an oven at 85 ${}^{\circ }$C for 10 min to partially dry the previous layers and ensure the pressure-sensitive layer did not stick to the mesh after printing, causing irregular layers.

**Figure 12:**
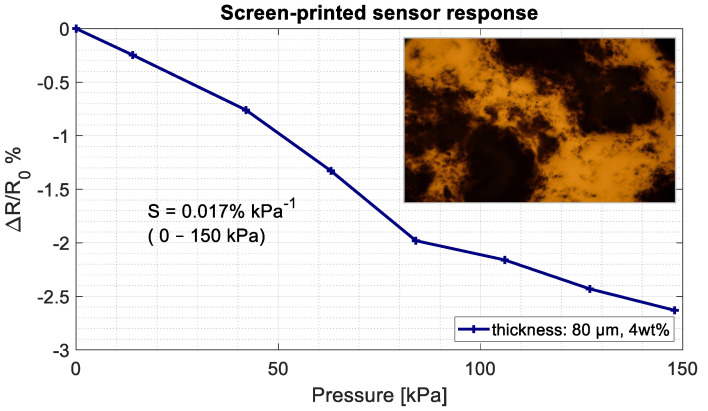
Screen-printed pressure sensor results. The resistance increases with the increasing pressure.

The resulting base-resistance was in the range of 400–600 Ω. As the pressure increased, the resistance consistently went up, indicating a negative piezoresistive effect (Figure 12). Hence, this gives a contradicting behaviour than what would be expected: an inverse relation between resistance and pressure. Additionally, the sensitivity is one order of magnitude lower than the simulations showed and a high surface roughness was measured.

A proposed explanation for this effect would be that conductive islands were formed on the surface due to the printed mesh-structure. When performing surface measurements, it was found that large gaps were present in the conductive polymer with height differences up to 40 μm. The suspicion was further confirmed when looking at the inset shown in Figure 12, depicting a microscopic image of large agglomerated CNTs with gaps in between and small amounts of CNTs in the polymer. The gaps correspond with the 50 μm mesh size. It is thought that with increasing pressure, the so-called conductive islands are pushed even further apart, decreasing conductivity.

### 4.2. 3D Mold-Printed Sensitive Layer

The 3D mold-printed layer was implemented due to the fact that the screen-printed method did not result in highly sensitive pressure-sensitive layers. A mold was printed with a thickness of 350 μm in which the PDMS/MWCNT solution was deposited and flattened by a squeegee (same device adopted as with screen-printing). The mold material was chosen to be PLA filament, as this material gives rigid structures and is easily available for 3D printers. Due to the temperature limitations of the mold material, the composite was cured at a maximum temperature of 70 ${}^{\circ }$C. It was found that the minimum 3D printing thickness was 150 μm, which yielded a dry-layer thicknesses of ≈100 μm.

**Figure 13:**
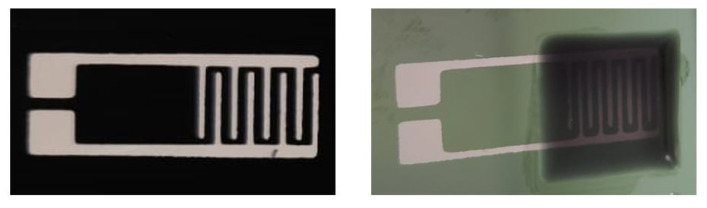
Screen-printed interdigital electrodes (left). CNT-PDMS composite mold-printed on top of electrodes (right). For clarification purposes a 0.2 CNT wt% solution was printed in order to leave the electrodes visible through the layer.

An advantage of this method was thought to be that the possibly agglomerated MWCNTs did not get stuck in the mesh of the screen but could be easily deposited with the mold. This method yielded thicker layers, but a minimum wet-layer of 150 μm was achievable. The layers were directly printed on the electrodes (see Figure 13) or printed separately and cut into the correct size, peeled of and placed on the IDE structures. For the latter, 50 μm anisotropic thick adhesive tape from SKS GmbH was applied to ensure adhesion.

**Figure 14:**
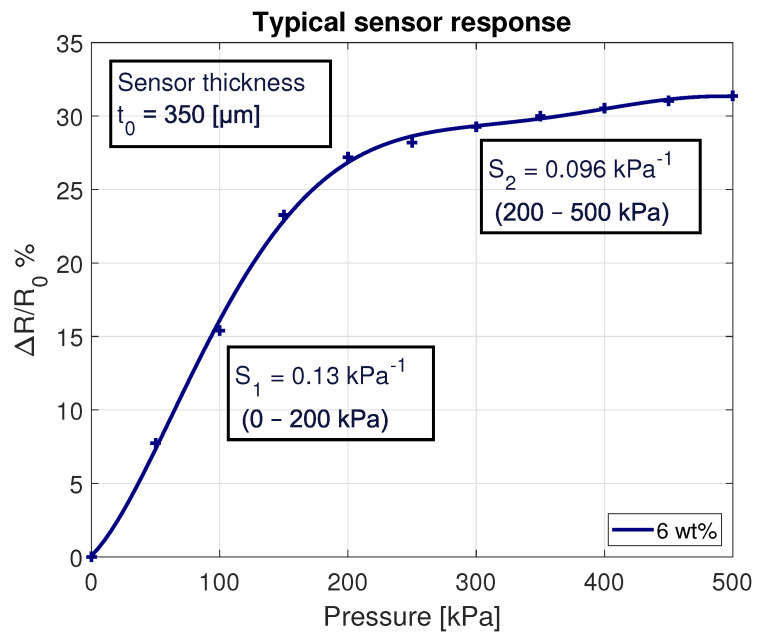
Typical sensor response with 6 wt% MWCNT, with a thickness of 350μm.

It was found that by placing the separate PDMS/MWCNT layer on the IDE structures, the first measurements were unstable. The sensor output showed the expected drop in resistance with applied pressure, but after relieving the pressure, the base resistance also dropped. After several loading cycles, the base resistance remained constant, and readings were repeatable. It is thought that, with the layer printed separately, the contact between the sensitive layer and IDE structures needs to solidify during several cycles. The conductivity increases until both layers are firmly connected. The averaged results of the sensors are depicted in Figure 14. Two clear regions can be identified, presenting similar sensitivity values as determined in the 2D model shown in Figure 6.

## 5. Discussion of Different Sensor Fabrication Methods

The pressure-sensitive layer was fabricated by mold-printing 100–350 μm layers on top of the IDE structures. The layer thickness depends on the minimum feature size of the 3D printer that prints the mold. It was determined that a stable 150μm layer could be printed with the BCN3D Sigma R19 Dual 3D printer, which resulted in a dry-layer thickness of ≈100μm. Typical sensor sensitivities up to a pressure of 200 kPa are 0.13% kPa${}^{-1}$, with the expected inverse relation between pressure and resistance. The elastic modulus increased with increasing the CNT wt% (from 3 (0 wt%) to 7.1 (4 wt%) MPa). However, all samples remained highly flexible and suitable for flexible electronics.

Several commonly available solvents were mixed with the CNTs to improve dispersion. Experimental results showed that without solvents, the ultrasonic treatment was not effective due to the high viscosity of the CNT/PDMS mixture. The non-toxic IPA solvent showed no visible CNT settling after 180 min (Figure 2), whereas the other solvents (for example, N-Butyl acetate and Toluene) already showed settling after 10 min. This corresponds with results found in [9], indicating relative poor dispersion of CNTs in Toluene. Due to the toxic nature of chloroform, this solvent was not considered (although it was found that this could even yield better CNT dispersions [9,10,25]).

Theoretical percolation thresholds were analytically and numerically determined and found to be lower than the experimental results. With agglomeration volume estimations and results from Li et al. [1], the theoretical and experimental results could be matched. A 10% agglomeration volume could increase the percolation threshold from 0.02 to 0.8 wt% [1]. The numerical model gives insight in the expected behaviour of the sensor. The theoretical sensitivity (0.12% kPa${}^{-1}$) corresponds to the experimental sensitivity (0.13% kPa${}^{-1}$) for a pressure region up to 200 kPa.

Direct screen-printing the sensitive layer on the IDEs gave a very low pressure sensitivity (0.017% kPa${}^{-1}$) with a negative piezoresistive effect. It is expected that due to the relative coarse mesh size, the CNTs clustered together and formed islands in the layer. For future research, finer mesh sizes with updated dispersion methods could make screen-printing feasible. An advantage would be the possibility to create even thinner sensing layers ≈ (20 μm) instead of the 100 μm mold-printed layers.

## 6. Conclusions

A working flexible pressure sensor in the range of 0 to 500 kPa with a sensitivity of 0.13% kPa${}^{-1}$ was demonstrated. The sensor is manufactured in the laboratory without the need for expensive cleanroom processes. The acquired sensitivities correspond to the numerical models, which confirmed the expected sensitivity of 0.12% kPa${}^{-1}$. Higher concentrations of carbon nanotubes were needed than in the theoretical calculations possibly due to the 10%${}_{volume}$ of agglomerated CNTs. The achieved sensitivities, compared to previous work [3,26,27] with carbon-black as a filler, evidence large improvements over the same pressure range, which had a sensitivity of 0.0012% kPa${}^{-1}$.

Screen-printed layers gave non-homogeneous dispersion of CNTs due to the chosen mesh structure. The concentrated carbon nanotubes agglomerated under the openings with low concentrations under the mesh structures, resulting in conductive islands forming in the polymer layer. Therefore, 3D mold-printing was implemented to circumvent the mesh structure and successfully print the sensitive layer directly on the electrodes.
